# Preparation of Copper-Decorated Activated Carbon Derived from *Platamus occidentalis* Tree Fiber for Antimicrobial Applications

**DOI:** 10.3390/ma15175939

**Published:** 2022-08-27

**Authors:** Thembisile Mahlangu, Iviwe Arunachellan, Suprakas Sinha Ray, Maurice Onyango, Arjun Maity

**Affiliations:** 1 Green Engineering Research Group, Department of Chemical Engineering, Faculty of Engineering and the Built Environment, Durban University of Technology, Durban 4000, South Africa; 2 DSI/CSIR Centre of Nanostructured and Advanced Materials, 1-Meiring Naude Road, Pretoria 0001, South Africa; 3 Department of Chemical, Metallurgical and Materials Engineering, Tshwane University of Technology, Pretoria 0001, South Africa; 4 Department of Applied Chemistry, University of Johannesburg, Doornfontein, Johannesburg 2028, South Africa

**Keywords:** activated carbon, nanocomposites, functional materials, copper, water treatment, antimicrobial properties

## Abstract

This study focuses on a greener approach to synthesizing activated carbon by carbonizing *Platamus occidentalis* tree fibers (TFSA) with 98% H_2_SO_4_ at 100 °C. The resulted TFSA was employed as an effective adsorbent for copper ions in aqueous media, yielding copper decorated TFSA (Cu@TFSA). The successful adsorption of copper onto the TFSA was proven through extensive characterization techniques. Herein, the TEM and XPS showed that copper nanoparticles were formed *in situ* on the TFSA surface, without the use of additional reducing and stabilizing agents nor thermal treatment. The surface areas of TFSA and Cu@TFSA were 0.0150 m^2^/g and 0.3109 m^2^/g, respectively. Applying the Cu@TFSA as an antimicrobial agent against *Escherica coli* ( *E. coli*) and *Salmonella* resulted in the potential mitigation of complex secondary pollutants from water and wastewater. The Cu@TFSA exhibited outstanding antimicrobial activity against *E. coli* and *Salmonella* in both synthetic and raw water samples. This demonstrated a complete growth inhibition observed within 120 min of exposure. The bacteria inactivation took place through the destruction of the bacteria cell wall and was confirmed by the AFM analysis technique. Cu@TFSA has the potential to be used in the water and wastewater treatment sector as antimicrobial agents.

## 1. Introduction

The global water crisis is perpetuated by the rise in urbanization, industrialization, and increased agricultural activity [1]. Pathogens can be transmitted to drinking water sources and the soil, thus posing a huge health risk to humans and the ecosystem [2,3]. It is, therefore, imperative to treat environmental and drinking water to prevent waterborne illnesses and detrimental effects on the ecosystem. Conventional disinfection processes include ultraviolet light [4], chlorination [5,6], and membrane filtration [7]. However, most of these technologies are reported to be stretched to their limits and barely comply with the increasingly stringent water-quality laws [8,9]. In some advances, complete microbial disinfection could not be achieved without the integration of UV, chlorination, and ozonation [10,11,12]. Furthermore, the generation of more complex secondary waste and harmful by-products during the treatment stage calls for a different and sustainable approach to water treatment.

Activated carbon (AC) has been used in numerous applications, including air purification, cosmetics, and, most significantly, in water purification, for the removal of organic and inorganic hazards from the environment [13,14]. Researchers have been increasingly looking at sustainable biomaterials to derive relatively inexpensive solutions [15,16]. However, carbon can also be a source of sustenance for microorganisms, which results in the formation of biofilms [17], further promoting the growth of microorganisms. It is, therefore, beneficial for the AC used for these diverse applications to have additional antimicrobial properties. To impart antimicrobial properties onto AC, metals have been coated and reduced onto the surface of AC [18]. Commonly used metals include copper, silver nanoparticles, and metal oxides, such as zinc oxide [19], which are loaded onto the inorganic support materials using various techniques and can be used together to remove contaminants and microorganisms from air and water sources.

The use of trace elements, such as copper and silver, as antimicrobial agents has re-gained tremendous interest [20]. Furthermore, the use of copper as a disinfectant and transportation medium for drinking water is not a foreign concept. Copper is also relatively less expensive than silver [21]. 

Copper has been used in various forms and on numerous AC supports, for water disinfection. Dankovich *et al*. incorporated copper nanoparticles (CuNPs) into paper by copper reduction using ascorbic acid and heat, for water disinfection [21]. Granulated activated carbon was impregnated with silver and copper oxide nanoparticles, with the aid of heat treatment (350 °C) for inactivating viruses in water [22]. Arakawa *et al*. impregnated silver–copper oxide nanoparticles on AC by subjecting them to vacuum (500 mmHg) and subsequent heat treatment (350 °C) [23]. The common method of imparting antimicrobial properties onto AC has been to immerse the AC in the metallic solution and use a combination of heat, microwave, chemical, and/or ultrasonic treatment as a way of reducing the copper. The use of additional treatment methods to ensure or increase disinfection performance and material stability comes with increased costs and increased complexities in synthesis. This leads to the generation of unwanted chemical waste. AC can also be regenerated using thermal and chemical treatments, which lead to additional chemicals that need to be discarded, increasing operational costs, too. 

This study reports on the re-use of a spent activated carbon adsorbent that was used for the removal of copper from aqueous media [24]. Also, the method of synthesis of the adsorbent further eliminates the need for additional chemical or heat treatment for the formation of copper nanoparticles on the surface of the activated carbon. The activated carbon was derived from tree fibers that were, subsequently, treated with sulfuric acid under moderate temperatures. The sulfuric acid-treated tree fiber (TFSA) loaded with copper nanoparticles via adsorption (Cu@TFSA) were used against *Escherica coli* (*E. coli*) and *Salmonella*. The TFSA and Cu@TFSA nanocomposites were characterized using techniques such as scanning electron microscopy (SEM), low-temperature N_2_ adsorption−desorption technique (BET), Fourier-transform infrared spectroscopy (FTIR), X-ray diffraction spectroscopy (XRD), and X-ray photon spectroscopy. The mechanism in which bacteria lysis takes place after exposure to Cu@TFSA was carried out using the atomic force microscope (AFM). 

## 2. Materials and Methods

### 2.1. Chemicals

Tree fibers were collected from the *Platamus occidentalis* tree, CSIR Campus, Pretoria, South Africa (25.7530° S, 28.2768° E). Sulfuric acid (98% H_2_SO_4_), sodium bicarbonate (1% *w/v* NaHCO_3_ solution), copper nitrate (Cu (NO_3_)_2_) and sodium thiosulfate (Na_2_S_2_O_3_) were purchased from Sigma-Aldrich, Johannesburg, South Africa. Sodium chloride (NaCl) was purchased from Minema Chemicals, Roodepoort, South Africa. Nutrient agar and nutrient broth were purchased from Merck, Lethabong, South Africa. Deionized water, collected from an EASYpure^®^ II, UV-ultrapure water system, was used for the preparation of all experimental solutions. All the other reagents were used as is unless otherwise stated.

### 2.2. Preparation of Sulfuric Acid Modified Tree Fiber (TFSA) Carbonized Material and Cu@TFSA NC

5 g of washed *Platamus occidentalis* tree fibers was carbonized by adding 98% H_2_SO_4_ (1:1) to the fibers and placed in the vacuum oven at 100 °C for 24 h. After carbonization, the material was dispersed washed with deionized water, under constant shaking, for 2 h to remove unreacted acid, then filtered through under suction. The carbonized material was soaked in 1% *w/v* NaHCO_3_ solution to ensure the complete removal of unreacted acid [24]. The copper-loaded TFSA (Cu@TFSA) was prepared via batch adsorption. A 1000 mg/L copper ions stock solution was prepared using Cu (NO_3_)_2_. The TFSA was immersed in 50 mL of a copper solution of a pre-determined concentration (5–200 mg/L) at 25 °C in a thermostatic shaker bath for 24 h. The material was then filtered under suction and dried at room temperature overnight. The Cu@TFSA was then used for antimicrobial testing. The residual copper ions concentration post-adsorption was determined using ICPMS (Thermo Scientific, Waltham, MA, USA).

### 2.3. Characterization of AC

The morphology of TFSA was investigated by field emission scanning electron microscope (FE-SEM, LEO Zeiss SEM, Oberkochen, Germany) operated at 3 kV and high resolution-transmission electron microscope (HR-TEM, JEOL JEM-2100, Tokyo, Japan) with a LaB6 filament operated at 200 kV. The chemical composition of TFSA, before and after copper adsorption, was determined on the spectrum-100 attenuated total reflectance-Fourier transform infrared (ATR-FTIR, Perkin-Elmer, Waltham, MA, USA). The crystallographic properties were performed on X-ray powder diffractometer with Cu anode (PANalytical Co. X’pert PRO, Worcestershire, UK). To determine the specific surface area of TFSA at low temperature, N_2_ adsorption–desorption technique was employed by using a Micromeritics TRISTAR II 3020 BET analyzer (Norcross, GA, USA). Surface charge measurements of the TFSA were made at various pH and evaluated using a Zeta-Sizer, Malvern Ltd., Malvern, UK. The thermal properties of the AC, before and after adsorption, were carried out on the TG-Q500 analyzer (TA Instruments, New Castle, DE, USA). Samples were heated from room temperature to 900 °C, with a heating rate of 10 °C/min in air atmosphere. X-ray photoemission spectroscopy (XPS) was performed using a SPECS PHOIBOS 150 hemispherical electron energy analyzer (Berlin, Germany) and a monochromatized Al Kα photon source (hν = 1486.71 eV). The overall energy resolution was set to 0.5 eV for all the spectra displayed in this study. The bactericidal mechanism of Cu@TFSA was confirmed by the VEECO Digital instruments Nanoscope, Multimode Atomic Force Microscope (Plainview, NY, USA). 

### 2.4. Antimicrobial Testing

Antimicrobial tests were conducted following a protocol published in our previous study [25]. Briefly, 1 mL and 0.9 mL aliquots of 15 % *w/v* sodium thiosulfate and 0.9% saline water were prepared, respectively. 100 mg of Cu@TFSA were suspended in 20 mL of sterile deionized water. Separate tubes were inoculated with *E. coli* (American Type Culture Collection^®^; ATCCR 25922, Manassas, VA, USA) and *Salmonella* to make up 6 × 10^3^ CFU/mL. Control experiments consisting of TFSA and bacteria, and bacteria solutions only, were also performed. All the experiments were carried out in triplicates, using different bacteria cultures, to get the average result. 0.1 mL samples were collected every 10 min for the first 60 min (starting from time 0) and every hour thereafter. The collected samples were added to the sodium thiosulfate aliquots, to deactivate the copper, and further diluted in 0.9% saline water. 0.01 mL of the deactivated samples was plated on nutrient agar using the drop plate technique [25]. The plates were incubated at 37 °C for 24 h. The surviving colonies were counted using an aCOLade 2 manual colony counter and the results were recorded in CFU/mL.

### 2.5. Bactericidal Mechanism of Cu@TFSA

The mechanism in which bacteria lysis takes place after exposure to Cu@TFSA was carried out using the AFM. The method of exposure was the same as that in Section 2.4 The samples were collected after 10 min of exposure and fixed for 2 h in 2.5% (*v/v*) glutaraldehyde. Thereafter, the samples were washed with 0.9% saline water, by centrifugation at 10,000 rpm for 1 min each cycle.

## 3. Results and Discussion

### 3.1. Characterization

**Figure 1:**
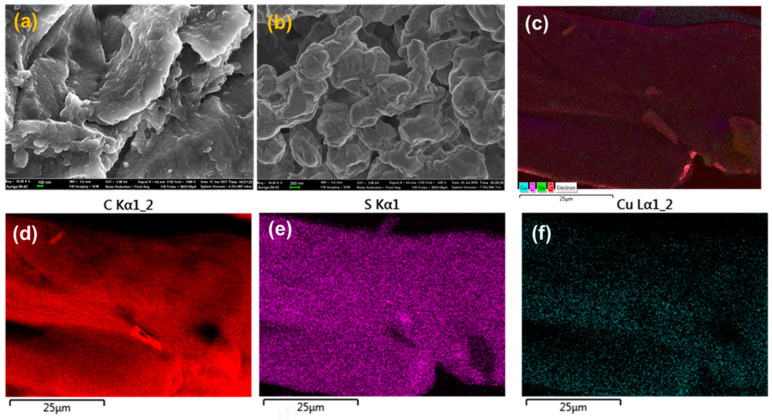
FE-SEM micrographs of (**a**) TFSA and (**b**) Cu@TFSA; with elemental mapping of (**c**) Cu, S, C, (**d**) C, (**e**) S, and (**f**) Cu.

The carbonized tree fiber (TFSA) and Cu-loaded carbonized tree fiber (Cu@TFSA) were successfully synthesized following acid treatment and carbonization at 100 °C and appeared as a black powder. The residual copper after adsorption was determined using the ICP-MS, Thermo Fisher Scientific. Thereafter, the copper loading onto TFSA was calculated to be 3.6 wt%. The morphology of the TFSA and Cu@TFSA was investigated using the FE-SEM and the micrographs are presented in Figure 1. The morphology of the TFSA (Figure 1a) appeared as smooth layer-like carbon sheets and following the adsorption of copper (Figure 1b), the morphology changed to thinner, meshed sheet-like structures. The elemental mapping (Figure 1c–f) and EDX spectra (Figure S1) confirmed the adsorption of copper onto the TFSA surface. 

**Figure 2:**
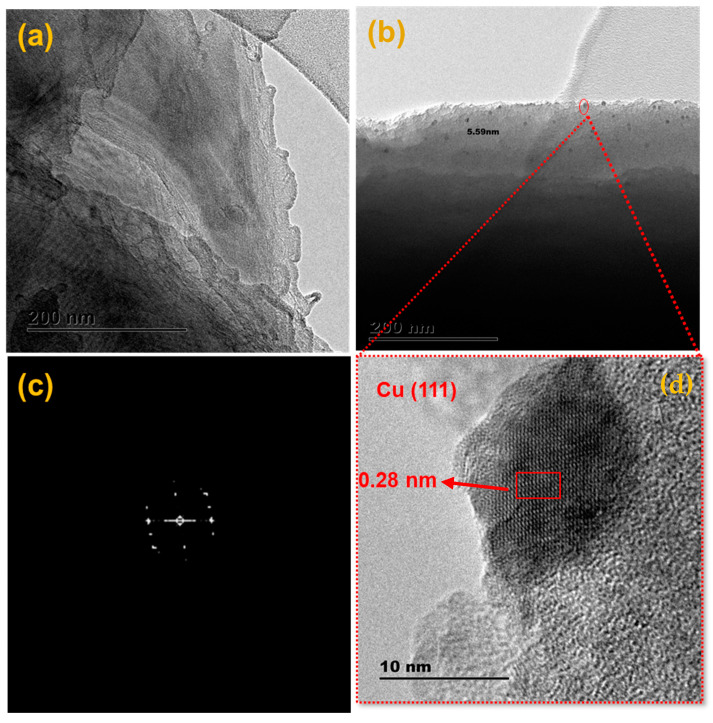
TEM images of (**a**) TFSA, (**b**,**d**) Cu@TFSA at two different magnifications, and (**c**) the corresponding FFT diffractogram of Cu@TFSA.

The surface characteristics were further investigated using HR-TEM (Figure 2). The sheet-like structures of the TFSA were observed once again presented in Figure 2a, with thinner layers observed in Figure 2b. The formation of Cu NPs was observed as black dots measuring 5.5 ± 06 nm on the TFSA surface were formed (Figure 2b). In the insert of Figure 2b, lattice fringes of the Cu NPs were observed, thus confirming their formation during or after adsorption of Cu^2+^ ions onto the surface of the TFSA. The d-spacing was measured to be 0.28 nm, which is attributed to the (111) plane of copper [26]. The crystallinity of the Cu@TFSA was also confirmed by the fast Fourier transform (FFT) diffractogram in Figure 2c [27]. The active presence of the (111) plane of the Cu NPs translates to the vital increase in antimicrobial efficiency of the nanocomposite. This phenomenon is attributable to the strong electrostatic interactions between the Cu NPs and the bacteria because of the active uncoordinated edge- and corner-atoms [28].

**Figure 3:**
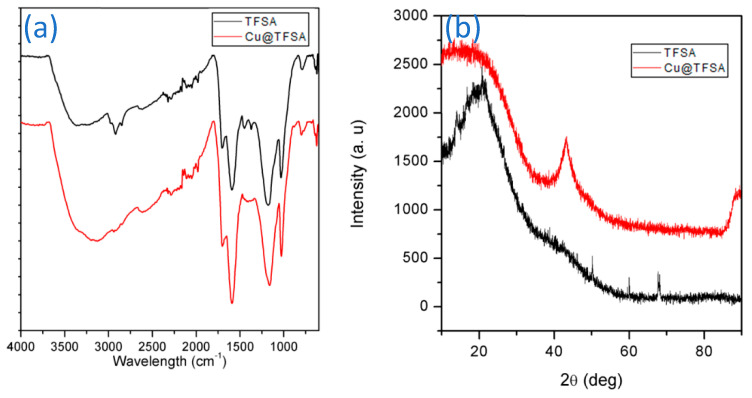
(**a**) ATR-FTIR spectra of TFSA before and after Cu^2+^ adsorption; and (**b**) XRD patterns of TFSA before and after Cu^2+^ adsorption (C_0_ = 150 mg/L, 25 °C, pH = 5.5).

To confirm the synthesis of TFSA and adsorption of Cu onto TFSA, the ATR-FTIR analysis was performed and presented in Figure 3a. The TFSA spectrum demonstrated peaks at 3367 cm^−1^ and 2920 cm^−1^, attributed to the –OH group and aliphatic –CH_2_ stretching vibrations, respectively. The C≡C stretching in alkyne groups is represented by the peak at 2322 cm^−1^. The peak at 1740 cm^−1^ is indicative of the C=O axial deformation of carboxylic groups, with the peaks at 1593 and 1179 cm^−1^ attributed to C–O stretching [29,30]. The Cu@TFSA spectrum was also presented in Figure 3a, where it was observed that the peaks at 1451 and 1372 cm^−1^ (COOH) had disappeared following Cu adsorption. Copper has an affinity for oxygen-containing groups; thus, it could serve as confirmation that the copper was adsorbed onto the TFSA through the COOH group [31]. A similar outcome was observed as shown by Shu *et al*., 2017 [30]. Furthermore, there was a shift in peak positions to the right, following copper adsorption, which also confirms that copper was adsorbed onto the TFSA surface. The XRD was used to further prove the adsorption of copper onto the TFSA surface. The XRD spectra of TFSA and Cu@TFSA are shown in Figure 3b. The graphitic curve of TFSA was observed with a few impurities observed after 2θ = 23° [32]. On the Cu@TFSA spectrum, a sharp peak was observed at 2θ = 45°, which is attributable to the (111) planar interface of Cu NPs [33,34]. 

**Figure 4:**
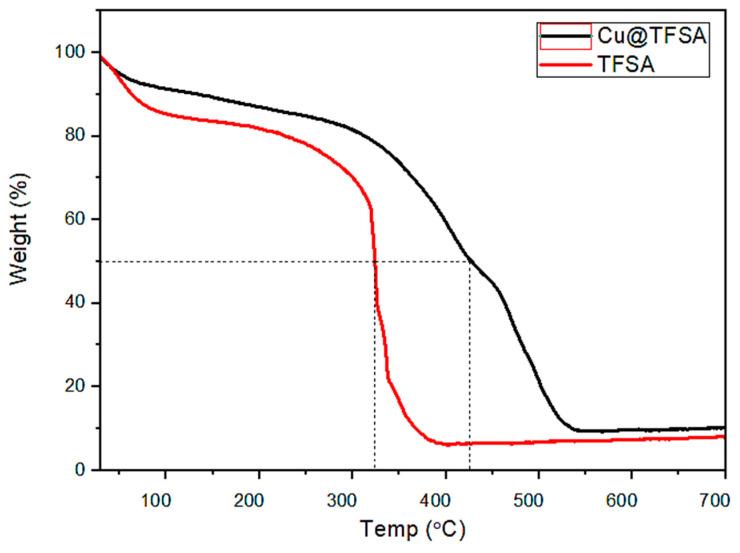
Thermogravimetric analysis of AC before and after Cu^2+^ adsorption.

The thermal properties of TFSA and Cu@TFSA were analyzed using Thermo gravimetric analysis (TGA) under air with heating a rate of 10 °C/min up to 900 °C. The weight loss observed at 100 °C was due to the loss of surface adsorbed moisture [35]. The thermal stability of TFSA and Cu@TFSA was analyzed at T_50%_, where 50% of the total loss has occurred and the results are shown in Figure 4. An increase in T_50%_ value from 305 to 405 °C was observed for TFSA and Cu@TFSA, respectively, thus indicating the presence of Cu in the Cu@TFSA material. These studies also indicated that the prepared Cu@TFSA nanocomposite is thermally stable and could be used at higher temperatures. This phenomenon proves that the CuO is not in its elemental form but is actual NPs. Carbon-based materials containing the elemental form of CuO seem to have accelerated degradation due to the catalytic effect of CuO on the oxidation of carbon [36]. 

The specific surface areas of TFSA and Cu@TFSA were 0.0150 m^2^/g and 0.3109 m^2^/g, respectively. The increase in surface area was indicative of the presence of Cu on the TFSA surface. Pore volumes were not obtainable using this technique due to very low porosity of the TFSA, as also seen in the FE-SEM images (Figure 1).

**Figure 5:**
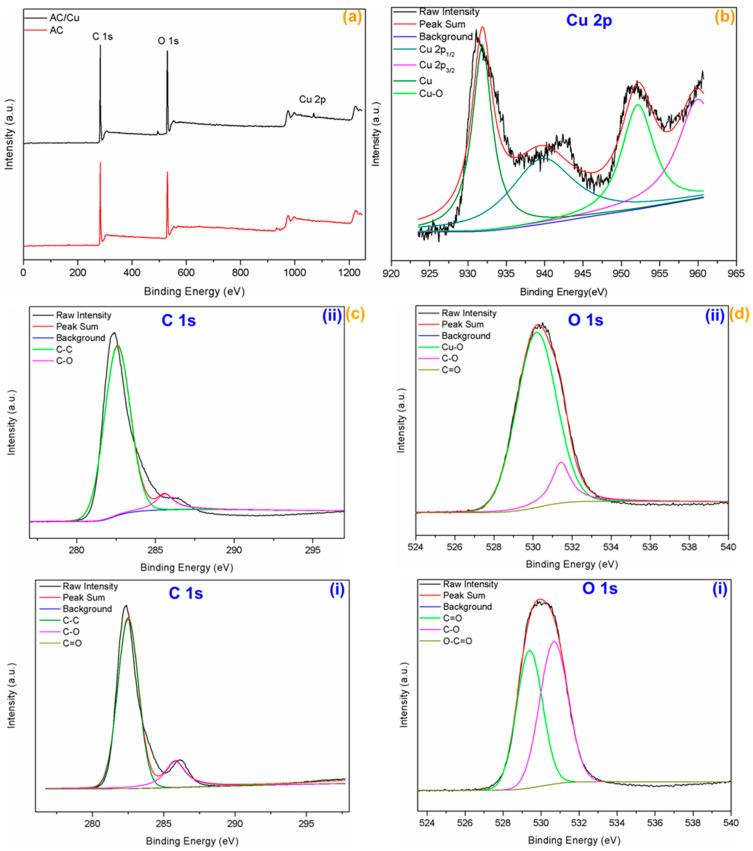
(**a**) XPS total spectral survey of TFSA (AC) and Cu@TFSA (Cu/AC); (**b**) high-resolution spectra of Cu 2p (**c**) C 1s, and (**d**) O 1s of (**i**) TFSA, and (**ii**) Cu@TFSA, respectively.

XPS measurements were performed to understand the way Cu^2+^ was adsorbed onto TFSA. The binding energies of C 1s, O 1s and Cu 2p were determined by means of the XPS analysis. The full survey scan spectra and high-resolution XPS spectra of the different elements present in TFSA and Cu@TFSA are presented in Figure 5. The survey scans spectra of TFSA and Cu@TFSA (Figure 5a) displayed the dominant peaks C 1s and O 1s peaks at 282 and 530 eV, respectively. After adsorption, the Cu 2p core level was observed, as expected. The successful adsorption of Cu^2+^ onto TFSA was also evident with the presence of the Cu 2p_1/2_ and Cu 2p_3/4_ (Figure 5b) at 932.8 and 953.9 eV, respectively [37]. Figure 5c displayed the C 1s binding energies representative of C-C, C-O, and C=O at 284.2, 285.6, and 288.0 eV, respectively [38]. The presence of Cu 2p_3/4_ was indicative of the presence of CuO [39], as well as the peak at 531.3 eV in O 1s spectrum shown in Figure 5(dii) [30]. Also, in Figure 5(di), a decrease in intensity of the C-O peak at 531.3 eV was observed, whilst there was increased intensity in the same peak in Figure 5(dii) attributable to CuO [30]. Given these results, there is a strong possibility that the Cu^2+^ was oxidised to form Cu NPs [40].

Overall, the characterization suggests that the copper was successfully adsorbed onto the TFSA surface. The TEM images show that the copper nanoparticles were formed on the surface. The disappearing COOH group on the FTIR and the presence of the CuO peaks on the XPS, suggest that the nanoparticles were formed *in situ*. Although there is no clear distinction between Cu(I) and Cu(0), all the supporting evidence suggests that the nanoparticles were formed during adsorption. This is corroborated by absence of catalytic degradation during the thermal degradation analysis. The adsorption kinetics were studied in detail in the preceding study [24]. Briefly, the adsorption of copper onto TFSA followed a pseudo-first-order model, thus copper was adsorbed homogeneously on the TFSA surface. The adsorption capacity was 11 mg/g.

### 3.2. Application of Cu@TFSA as an Antimicrobial Agent

Cu@TFSA was tested against *E. coli* (American Type Culture Collection^®^; ATCCR 25922) and *Salmonella*. TFSA and water without TFSA were used as controls, to ensure that the microbicidal effect was exclusively from the Cu@TFSA. Cu^2+^ adsorption using TFSA was carried out at varying concentrations of 50 mL Cu^2+^ solutions (10–200 mg/L) in 100 mL glass bottles, at 200 rpm and 25 °C in a thermostatic shaking water bath (Separations Scientific, Roodepoort, South Africa) for 24 h. The Cu@TFSA was dried at room temperature overnight, ground and stored for further use. 100 mg of Cu@TFSA were dispersed in 20 mL sterile ultrapure water and inoculated with *E. coli* to make up a final concentration of 1 × 10^7^ CFU/mL. The exposure time of the *E. coli* to the Cu@TFSA was kept at 60 min. The effect of Cu^2+^ loading is presented in Figure S2. It was observed that the Cu@TFSA loading from the initial Cu^2+^ concentration of 150 mg/L, possessed the highest killing effect (Table S1).

**Figure 6:**
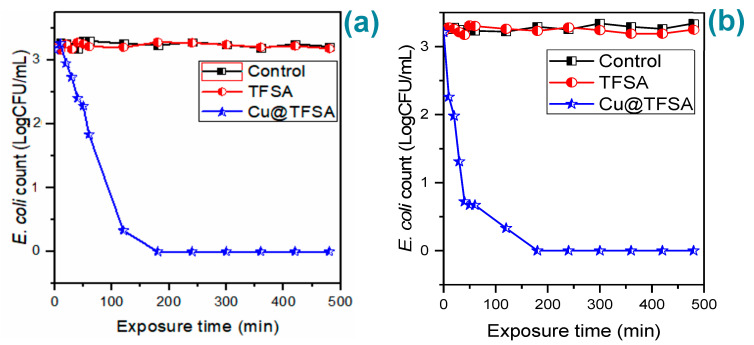
The effect of exposure time on *E. coli* by Cu@TFSA in river water (**a**) and (**b**) the effect of exposure time on *E. coli* by Cu@TFSA in groundwater.

The ideal Cu^2+^ concentration that was chosen to for the continuation of the study was 150 mg/L, due to the Cu@TFSA loaded material (3.6 wt% of Cu loaded) for this concentration being the most efficient for water disinfection in 60 min. Further antimicrobial studies were performed with river and groundwater to determine the bactericidal competence of Cu@TFSA in real water samples. The exposure time was extended to 480 min for this aspect of the study and the initial *E. coli* concentration was 6 × 10^3^ CFU/mL. The characteristics of the real field samples are summarized: River water-pH: 7.10 ± 0.01, total coliform count: 390 CFU/100 mL and *E. coli*: 38 CFU/100 mL; groundwater (borehole)–pH: 6.40 ± 0.01, total coliform count: 0 CFU/100 mL, and *E. coli*: 0 CFU/100 mL. The water was sterilized prior to use and inoculated with *E. coli* to the desired concentration. The disinfection efficacy of Cu@TFSA in river and groundwater is presented in Figure 6a,b, respectively. A similar trend was observed for both river and groundwater, whereby there was a steady decrease in bacterial growth until complete growth inhibition was observed at 180 min. The re-growth of *E. coli* was tested by plating samples at 1440 min time point, and no re-growth was observed.

**Figure 7:**
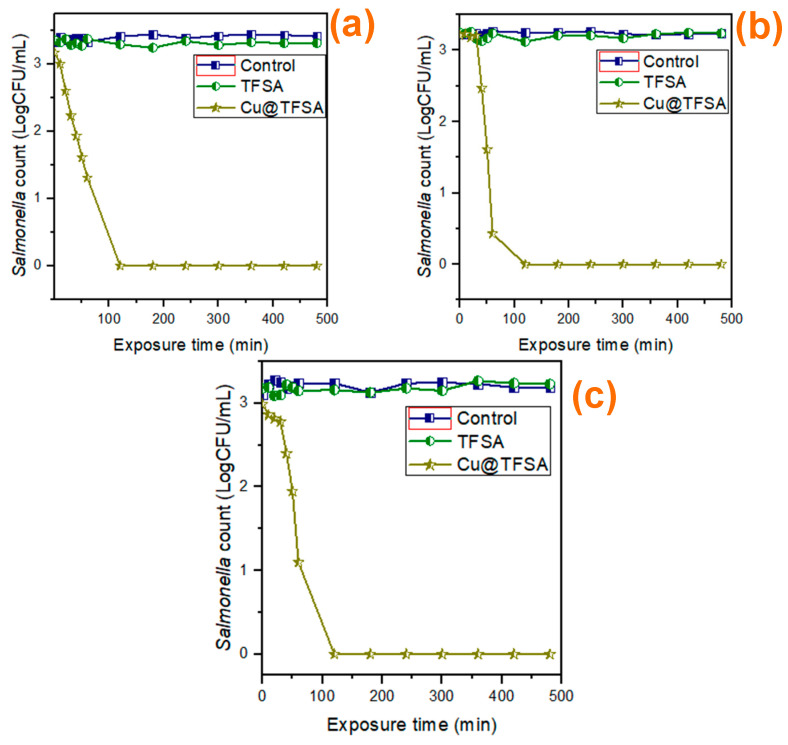
(**a**) The effect of exposure time on *Salmonella* by Cu@TFSA in synthetic water; (**b**) the effect of exposure time on *Salmonella* by Cu@TFSA in groundwater, and (**c**) the effect of exposure time on *Salmonella* by Cu@TFSA in river water.

The antimicrobial effect of Cu@TFSA was also tested against *Salmonella.* The experimental conditions were kept the same at 100 mg Cu@TFSA in 20 mL water and 480 min exposure time. The antimicrobial effect of Cu@TFSA on *Salmonella* initially tested in sterile synthetic water (Figure 7a) and complete growth inhibition was observed at 120 min.

Again, sterile river and groundwater were used as real water samples. The results for river and groundwater are shown in Figure 7a,b, respectively. A steady decrease in *Salmonella* concentration was observed until complete growth inhibition was seen at 120 min, for both water sources, which meant that Cu@TFSA performed similarly, if not the same, for *Salmonella* disinfection. Also, at 1440 min, there was no bacteria growth observed. Thus, the Cu@TFSA is indeed killing the bacteria rather than just inhibiting the growth.

### 3.3. E. coli Inactivation Kinetics by Cu@TFSA

The inactivation kinetics of *E. coli* were investigated using the pseudo-first-order kinetics equation given below. The samples with an initial Cu^2+^ concentration of 100 mg/L, 150 mg/L and 200 mg/L were considered.

**Figure 8:**
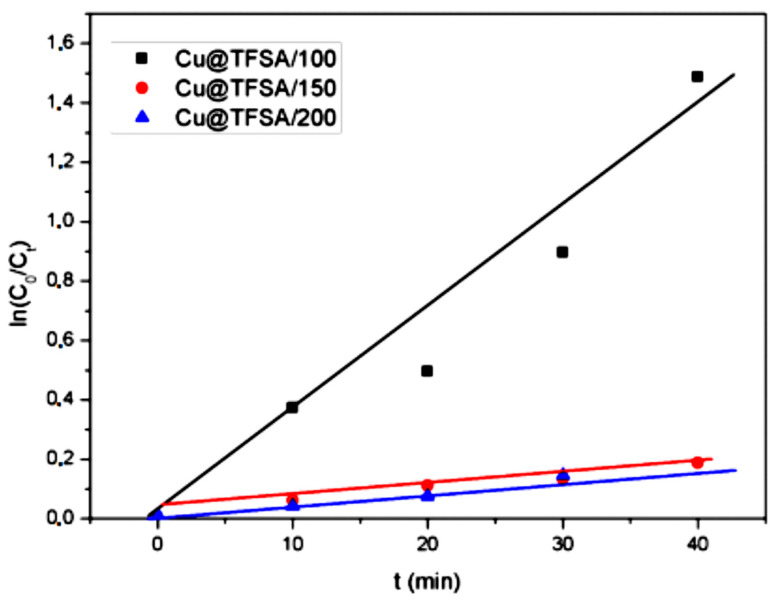
The plot of ln(C_0_/C_t_) vs. time, t, varying the copper loading on the TFSA.

Ln(C_t_) = −*k*t + ln(C_0_) (1)

where C_0_ and C_t_ are the initial *E. coli* concentration and *E. coli* concentration at time, t, respectively. The rate constant, *k*, is determined from the linear regression curves in Figure 8. The disinfection of *E. coli* followed the behavior of the pseudo-first-order kinetics behavior. The *k* values for copper loading from 100, 150, and 200 mg/mL were 0.2516 m^−1^, 0.0635 m^−1^, and 0.0428 m^−1^, respectively. The rate decreases as the copper loading is increased, whilst the antimicrobial activity increased with increasing copper loading. As the initial concentration decreases, so does the rate. However, at a higher copper loading, the faster the bacteria disinfection takes place. Thus, there is a reduction in reaction rate [41,42].

### 3.4. Antibacterial Mechanisim

**Figure 9:**
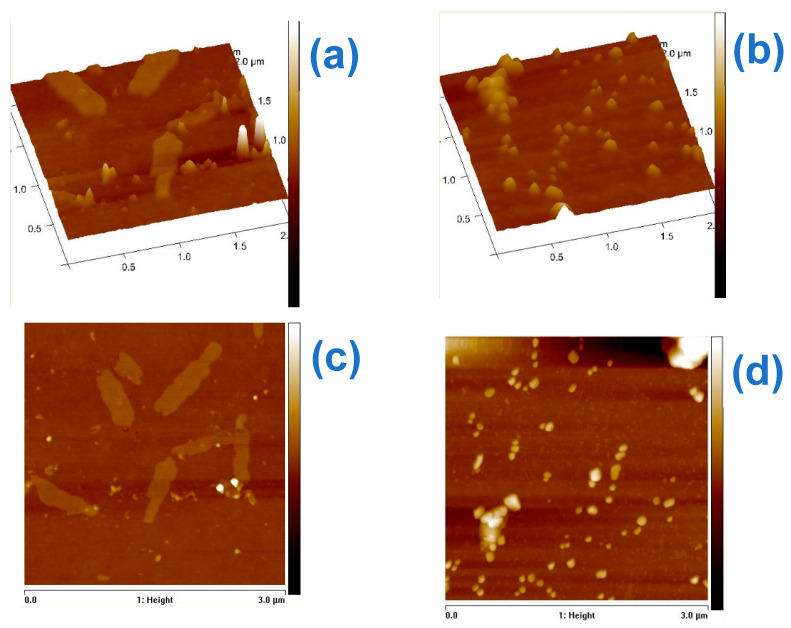
AFM images of control *E. coli* samples (**a**) 3D and (**c**) 2D-height; and *E. coli* cells after 20 min of exposure to Cu@TFSA (**b**) 3D and (**d**) 2D-height.

To develop a suitable point-of-use device, the mechanism of how the bacteria is killed is of major importance. The mechanism was investigated using the AFM and shown in Figure 9. As seen in Figure 9a,c, the *E. coli* cells were intact, and the rod shape was retained. However, after exposure to the Cu@TFSA, fragments of the cell were observed, indicating that the cell walls were ruptured and disintegrated (Figure 9b,d). The microbicidal mechanism is described to be multimodal. The synergistic *E. coli* inactivation of the Cu NPs and TFSA was evident in the extent of damage that was done to the *E. coli* cell wall. Cu NPs are reported to cause numerous toxic effects that lead to the formation of cell fragments [43,44]. Also, using TFSA as a support for the Cu NPs, increases the chances of contact between Cu NPs and the microbes, due to an increased surface area which, in turn, increases the bioavailability of Cu [19,27]. Since Cu NPs have a strong reduction ability, the redox cycles between Cu^0^, Cu^1+^, and Cu^2+^ mediate the generation of reactive oxygen species; thus, inducing cell death [21]. Cu^2+^ and Cu^1+^ ions eluted from Cu NPs can be absorbed by bacteria, thus imparting damage to the cell membrane by solidifying protein structures or altering enzyme function, further leading to cell death [45,46]. It is also suggested that Cu^2+^ ions can penetrate the cell membrane upon combining with the plasma membrane during electrostatic interactions. The cell wall of gram-negative bacteria, consists of anionic surfaces (lipopolysaccharide molecules) that have a high affinity to the positively charged Cu^2+^, thus leading to increased toxicity [46]. More so, nanoparticles become a focal source of ions upon contact with the bacteria cell, because the dissolution takes place in the localized area [47]. This would lead to opening or closing of the membrane channels, altering the permeability of the membrane, causing intracellular ions to leak. Similarly, the Cu ions bond with intracellular amino acids and protease, leading to the protein denaturation and consequential cell death [44,48,49]. Cu ions also form hydroxyl free radicals that are prone to damaging proteins. However, hydrogen peroxide induces the release of Cu ions. This leads to increased toxicity because of the increased protein oxidation which further affects the gene expression and disruption of homeostasis The mechanism is shown in Equation (2) [48],

2 Cu^+^ + 2H^+^ +O_2_ → 2Cu^2+^ + H_2_O_2_ (2)

**Figure 10:**
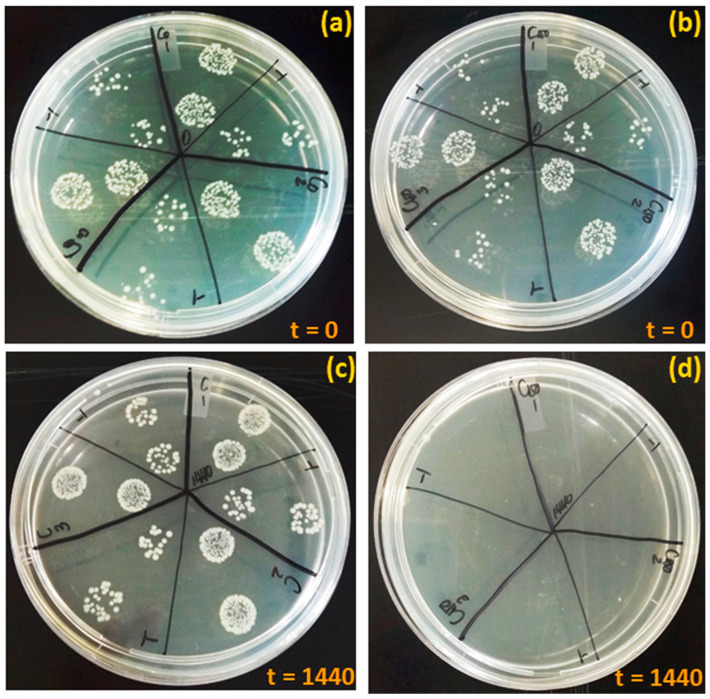
Pictorial diagram displaying the regrowth tests performed after exposure of *E. coli* to TFSA controls at (**a**) t = 0 and (**c**) t = 1440; regrowth tests performed after *E. coli* exposure to Cu@TFSA at (**b**) t = 0 and (**d**) t = 1440.

To justify the bactericidal mechanism, regrowth tests were done, and the results are displayed in Figure 10. The samples were re-plated after 24 h of sampling, to confirm that the *E. coli* cells were certainly inactivated. It was seen that the controls (C_0_ and C) still had viable cells at time points 0 and 1440 min, indicating that the cell death was not taking place naturally (Figure 10a–c). Hence there was no cell growth observed in Figure 10d, after the *E. coli* cells were exposed to Cu@TFSA (C_150_). 

The current work was compared to several studies, to gauge relevance and performance (Table 1). Most of the studies in the literature focussed on the effectiveness of the materials and not on how rapid the disinfection was. Therefore, the comparison was limited to the number of bacteria inactivated at the 24 h time point. This study has shown the superiority of the Cu@TFSA because there was rapid disinfection of bacteria at low doses of Cu.

### 3.5. Leaching Tests

Testing the extent of Cu^2+^ leaching post antimicrobial experiments was imperative. Therefore, leaching studies were conducted to quantify how much of the Cu^2+^ was leaching into the aqueous media. Disinfected synthetic and real water samples were filtered using a 0.45 µm syringe filter and the supernatants were tested for residual Cu^2+^ using the ICP-MS. Cu^2+^ ions were undetectable in the supernatants (even at samples taken after 24 h), thus indicating that the leaching was negligible and that the Cu NPs were rather stable. 

## 4. Conclusions

In this study, activated carbon was successfully derived from *Platamus occidentalis* tree fibers, through acid treatment at a moderate temperature. The activated carbon was decorated *in situ* with Cu NPs via copper ion adsorption, as revealed by HR-TEM, XRD, and XPS analyses. Upon adsorption, the Cu NPs formation was spontaneous. Thus, eliminating the use of reducing and/or stabilizing agents and thermal treatments, which is a step in the right direction for green synthesis practices. This method of synthesis could save on operational costs because of the reduction in synthesis steps and omission of the reducing agent. The increased surface area (TFSA-0.0150 m^2^/g and Cu@TFSA-0.3109 m^2^/g) was indicative of Cu adsorption. There was a significant change in morphology, after copper adsorption, as shown in the SEM. Also, the introduction of Cu enhanced the thermal properties of the nanocomposite with an increased T_50%_ of 100 °C. 3.6 wt% of copper was adsorbed onto the surface, which corresponds with the low porosity and surface area measurements obtained from the BET. However, the Cu@TFSA still exhibited excellent antimicrobial activity, whereby 4.9 log inactivation of *Salmonella* and *E. coli* were lysed, respectively. There was no bacteria re-colonization observed, which was indicative of the stability of the Cu NPs on the TFSA. The antimicrobial properties of the Cu@TFSA were solely attributed to the presence of Cu NPs on the TFSA surface. The kinetic behavior of the inactivation process followed a pseudo-first-order reaction model. Cu@TFSA would make a strong contender for water and drinking water disinfection applications because of the prolonged thermal and chemical stability. 

## Figures and Tables

**Table 1:** Comparative table of *E. coli* inactivation using various metal nanoparticle-based materials.

Material	Further Reduction and Treatment	Exposure Time (h)	Log Inactivation	Ref.
Ag_2_O-PAN	-	8	3	[50]
copper and zinc-doped hydroxyapatite	none	4	2	[51]
Cu/Sepiolite	90% Ar/10% H_2_, 500 °C	24	4.40	[52]
AgNP-AC	Tri-sodium citrate, 100 °C	-	4	[53]
CuHAp	none	24	2.67	[51]
Ag-AC	monohydrate and azote gas	-	2	[17]
CuO-filter	-	1	4.69	[54]
KOH-Cu-AC	90 °C/L-ascorbic acid/KOH	0.42	2	[55]
Cu@AC	none	2	4.9	This work

## Data Availability

Not applicable.

## Supplementary Materials

The following supporting information can be downloaded at: https://www.mdpi.com/article/10.3390/ma15175939/s1, Figure S1: EDX Spectra of (**a**) TFSA and (**b**) Cu@TFSA; Figure S2: Optimization of antimicrobial Cu@TFSA copper loading during exposure to *E. coli* samples; Table S1: The killing efficiency of Cu/AC with varying Cu^2+^ concentration at 60 min exposure time and *E. coli* concentration of 1 × 10^7^ CFU/mL.
