# Supplementary material for: Preparation of Copper-Decorated Activated Carbon Derived from Platamus occidentalis Tree Fiber for Antimicrobial Applications
Source: Materials (Basel). 2022 Aug 27;15(17):5939. doi: 10.3390/ma15175939 (PMC9457392; doi:10.3390/ma15175939)
Supplement: Supplementary file 1 [file materials-15-05939-s001.zip › materials-1844188-supplementary.pdf]

materials

Supporting Information

Preparation of Copper-Decorated Activated Carbon Derived from *Platamus occidentalis* Tree Fiber for Antimicrobial Applications

Thembisile Mahlangu [1,2,3,*], Iviwe Arunachellan [4], Suprakas Sinha Ray [2], Maurice Onyango [3] and Arjun Maity [2,3]

1 Green Engineering Research Group, Department of Chemical Engineering, Faculty of Engineering and the Built Environment, Durban University of Technology, Durban 4000, South Africa
2 DSI/CSIR Centre of Nanostructured and Advanced Materials, 1-Meiring Naude Road, Pretoria 0001, South Africa
3 Department of Chemical, Metallurgical and Materials Engineering, Tshwane University of Technology, Pretoria 0001, South Africa
4 Department of Applied Chemistry, University of Johannesburg, Doornfontein, Johannesburg 2028, South Africa
* Correspondence: thembisilem1@dut.ac.za; Tel.: +27-313732646

Table of contents

Page

Figure. S1. EDX Spectra of a) TFSA and b) Cu@TFSA S3

Figure S2: Optimization of antimicrobial Cu@TFSA copper loading during exposure to *E. coli* samples S4

Table S1: The killing efficiency of Cu@TFSA with varying Cu^{2+} concentration at 60 min exposure time and *E. coli* concentration of $1x10^7$ CFU/mL S4

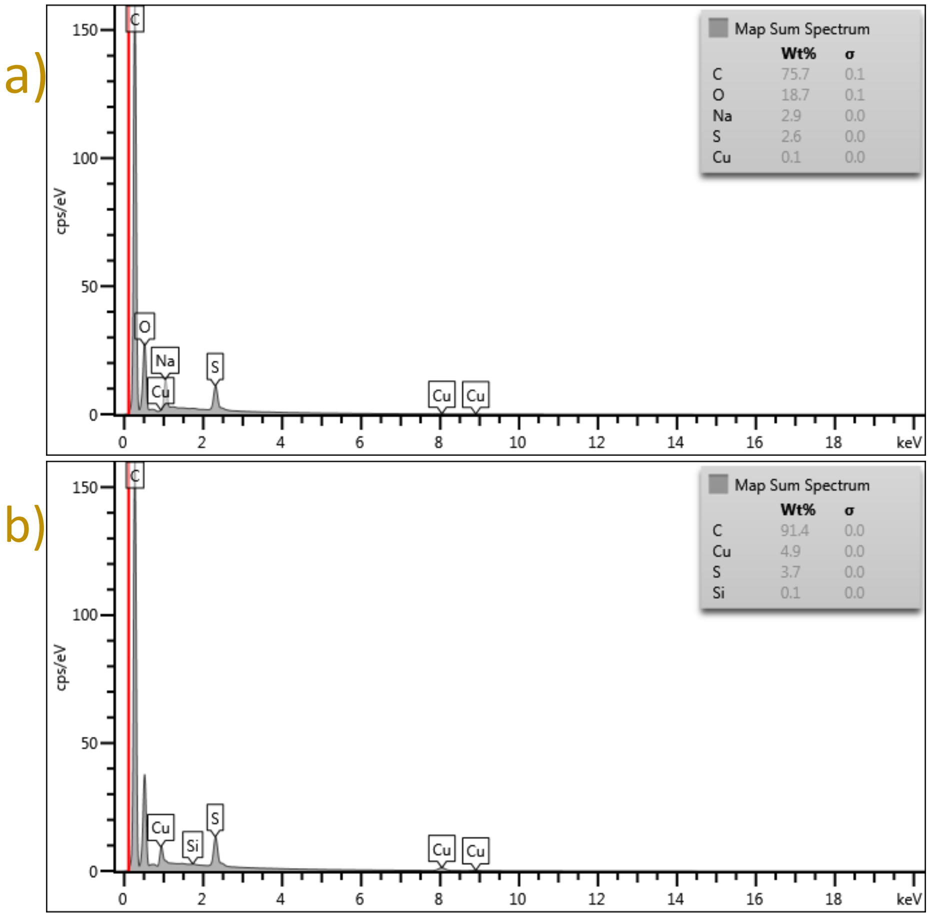

Figure S1: EDX Spectra of a) TFSA and b) Cu@TFSA.

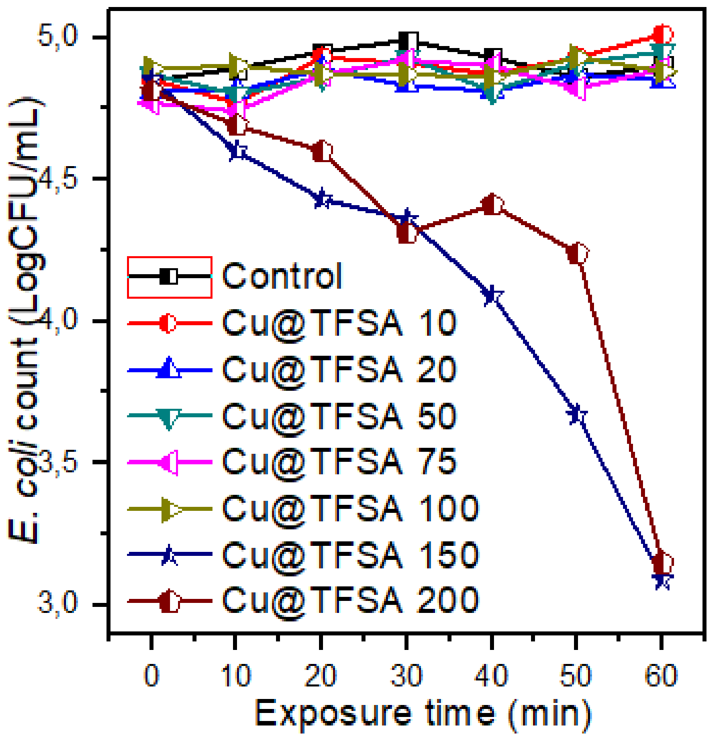

Figure S2: Optimization of antimicrobial Cu@TFSA copper loading during exposure to *E. coli* samples.

Table S1: The killing efficiency of Cu@TFSA with varying Cu^{2+} concentration at 60 min exposure time and *E. coli* concentration of 1x10^7 CFU/mL.

**Cu^{2+} concentration (mg/L)**	**Killing Efficiency (%)**
10	0
20	2.26
50	3.25
75	1.28
100	2.61
150	38.37
200	37.12
